# Association of Longitudinal β-Amyloid Accumulation Determined by Positron Emission Tomography With Clinical and Cognitive Decline in Adults With Probable Lewy Body Dementia

**DOI:** 10.1001/jamanetworkopen.2019.16439

**Published:** 2019-12-02

**Authors:** Zuzana Nedelska, Christopher G. Schwarz, Timothy G. Lesnick, Bradley F. Boeve, Scott A. Przybelski, Val J. Lowe, Walter K. Kremers, Jeffrey L. Gunter, Matthew L. Senjem, Jonathan Graff-Radford, Tanis J. Ferman, Julie A. Fields, David S. Knopman, Ronald C. Petersen, Clifford R. Jack, Kejal Kantarci

**Affiliations:** 1 Department of Radiology, Mayo Clinic, Rochester, Minnesota; 2 Department of Health Sciences, Mayo Clinic, Rochester, Minnesota; 3 Department of Neurology, Mayo Clinic, Rochester, Minnesota; 4 Department of Psychiatry and Psychology, Mayo Clinic, Jacksonville, Florida; 5 Department of Psychiatry and Psychology, Mayo Clinic, Rochester, Minnesota

## Abstract

**Importance:**

In patients with probable dementia with Lewy bodies (DLB), overlapping Alzheimer disease pathology is frequent and is associated with faster decline and shorter survival. More than half of patients with DLB have elevated β-amyloid levels on carbon-11 labeled Pittsburgh compound B (PiB) positron emission tomography, but the trajectory of longitudinal β-amyloid accumulation and its associations with clinical and cognitive decline in DLB are not known.

**Objectives:**

To determine the trajectory of β-amyloid accumulation in patients with probable DLB and to investigate the associations of β-amyloid accumulation with measures of clinical and cognitive decline over time in DLB.

**Design, Setting, and Participants:**

This cohort study included 35 consecutive patients with probable DLB from the Mayo Clinic Alzheimer Disease Research Center and matched them by age, sex, and apolipoprotein e4 status with 140 cognitively unimpaired participants from the population-based Mayo Clinic Study of Aging. Participants were observed from April 2010 to September 2017. Data analysis was conducted from January 2018 to January 2019.

**Exposure:**

Baseline and follow-up PiB positron emission tomography and comprehensive clinical evaluations.

**Main Outcomes and Measures:**

Rate of change in PiB standardized uptake value ratios (SUVRs) by PiB SUVR and time in years; the associations between baseline PiB SUVR, change in PiB SUVR, and change in several measures of clinical and cognitive decline.

**Results:**

A total of 175 participants were evaluated (35 [20.0%] with probable DLB; mean [SD] age, 69.6 [7.3] years; 16 [45.7%] apolipoprotein e4 carriers; 31 [88.6%] men; and 140 [80.0%] cognitively unimpaired adults; mean [SD] age, 69.7 [7.2] years; 64 [45.7%] apolipoprotein e4 carriers; 124 [88.6%] men). In both groups, the rates of change in PiB SUVR showed an initial acceleration at lower baseline PiB SUVR followed by a deceleration at higher baseline PiB SUVR, thus forming an inverted-U shape. The trajectories of the rates of change in PiB SUVR did not differ between participants with probable DLB and cognitively unimpaired participants in terms of shape (*P* = .59) or vertical shift (coefficient [SE] 0.007 [0.006]; *P* = .22). The integral association of cumulative PiB SUVR with time in years showed a sigmoid-shaped functional form in both groups. In participants with probable DLB, higher baseline PiB SUVR and change in PiB SUVR were associated with more rapid clinical decline, as measured by the Clinical Dementia Rating, sum of boxes (baseline PiB SUVR: regression coefficient [SE], 1.90 [0.63]; *P* = .005; *R*^2^ = 0.215; change in PiB SUVR, regression coefficient [SE], 16.17 [7.47]; *P* = .04; *R*^2^ = 0.124) and the Auditory Verbal Learning Test, delayed recall (baseline PiB SUVR, regression coefficient [SE], −2.09 [0.95]; *P* = .04; *R*^2^ = 0.182; change in PiB SUVR, regression coefficient [SE], −25.05 [10.04]; *P* = .02; *R*^2^ = 0.221).

**Conclusions and Relevance:**

In this study, the rate of change in PiB SUVR among participants with probable DLB increased, peaked, and then decreased, which was similar to the trajectory in cognitively unimpaired participants and the Alzheimer disease dementia continuum. Higher baseline PiB SUVR and change in PiB SUVR were associated with more rapid clinical and cognitive decline over time. Measuring the change in PiB SUVR has implications for designing anti–β-amyloid randomized clinical trials for individuals with probable DLB.

## Key Points

**Question:**

What is the trajectory of β-amyloid accumulation over time, and how is it associated with clinical and cognitive decline among patients with probable dementia with Lewy bodies?

**Findings:**

This cohort study of 175 participants found that the cumulative density of β-amyloid accumulation by time in years followed a sigmoid-shaped form among patients with probable dementia with Lewy bodies as well as among cognitively unimpaired participants who were matched by age, sex, and apolipoprotein e4 status. In dementia with Lewy bodies, both baseline and longitudinal β-amyloid load accumulation were associated with measures of clinical and cognitive decline over time.

**Meaning:**

The results of this study suggest that longitudinal β-amyloid accumulation among patients with dementia with Lewy bodies could be used to track the clinical progression of dementia with Lewy bodies and potentially to design clinical trials targeting β-amyloid in dementia with Lewy bodies.

## Teaser

This cohort study determines the trajectory of β-amyloid accumulation among patients with probable dementia with Lewy bodies and investigates the associations of β-amyloid accumulation with measures of clinical and cognitive decline over time in dementia with Lewy bodies.

## Introduction

Dementia with Lewy bodies (DLB) is a common neurodegenerative dementia associated with Lewy body disease pathology. Patients with probable DLB frequently have varying levels of Alzheimer disease (AD) pathology, β-amyloid, and neurofibrillary tangles (NFT), in addition to Lewy body disease pathology.^1,2^ In DLB, concomitant AD pathology has been associated with a faster clinical progression and a shorter survival in autopsy-confirmed cohorts.^3,4,5,6,7^

Positron emission tomography (PET) imaging with carbon-11 labeled Pittsburgh compound B (PiB) is a well-established biomarker of β-amyloid in vivo.^8,9,10^ Approximately two-thirds of patients with DLB have elevated PiB uptake on PET.^11^ However, the association of a higher PiB uptake with greater clinical or cognitive impairment has been equivocal in DLB cross-sectionally.^12^ Longitudinal studies in DLB are needed to understand the trajectory of PiB uptake over time and to determine its association with clinical progression. Monitoring these aspects will be important for identifying the most eligible candidates for emerging targeted treatments and for assessing the response to such treatments.

Using serial PiB PET, prospective studies^13,14,15^ in cognitively unimpaired (CU) and in cognitively impaired individuals within the AD continuum with a range of baseline PiB standardized uptake value ratios (SUVRs) demonstrated that the rate of change in PiB SUVR is not linear. At lower baseline PiB SUVR, the rate of change in PiB SUVR accelerates and then decelerates at a higher baseline PiB SUVR,^13,14,15^ thus forming an inverted-U shaped curve as a function of baseline PiB SUVR.^13,15^ Consequentially, cumulative PiB SUVR as a function of time follows a sigmoid-shaped trajectory,^13,15^ reaching a plateau at high baseline PiB SUVR within the AD continuum,^13,15^ with implications for the timing of treatment strategies.

In DLB, the trajectory of the change in PiB SUVR is not known. Nor is it known whether accelerated rates of change in PiB SUVR are associated with faster clinical declines in DLB. In this longitudinal PiB PET cohort study, our objective was to determine the change in PiB SUVR and the cumulative PiB SUVR over time in patients with probable DLB compared with CU adults with similar demographic characteristics. Our second objective was to evaluate the associations of baseline PiB SUVR and change in PiB SUVR with measures of longitudinal clinical and cognitive decline in probable DLB. A final objective was to calculate sample size estimates for a hypothetical randomized clinical trial targeting β-amyloid in DLB.

## Methods

### Data Source, Study Design, and Population

The probable DLB group included 35 consecutive patients observed through the Mayo Clinic Alzheimer Disease Research Center between April 2010 and September 2017, of whom 32 met clinical criteria for probable DLB at baseline^16^ and 3 had mild cognitive impairment (MCI) at baseline and developed probable DLB by the first follow up. To compare the trajectory of change in PiB SUVR, we included 140 CU participants observed through the Mayo Clinic Study of Aging, a longitudinal, population-based cohort study.^17^ Cognitively unimpaired individuals were matched 4:1 with patients with probable DLB on age, sex, and apolipoprotein (*APOE*) e4 status; they remained CU throughout the study duration.

### Baseline and Follow-up Visits

All participants were required to have a baseline PiB PET coupled with a comprehensive clinical evaluation and an identical follow-up within 12 to 15 months for the probable DLB group and within 15 to 30 months for the CU group. Baseline and follow-up visits incorporated a medical history review, informant interview, neurologic examination, neuropsychological assessment, and a series of informant questionnaires.^3,17,18,19^ After each visit, a consensus panel, composed of the study nurse, neurologist (B.F.B, J.G.-R., D.S.K., or R.C.P.), and neuropsychologist (T.J.F. or J.A.F.) who evaluated the participant, established the clinical diagnosis after accounting for visual or hearing deficits, education, and prior level of functioning.

### Clinical and Cognitive Measures

Clinical severity and progression were determined using global cognitive assessments (ie, Mini-Mental State Examination [MMSE] and Dementia Rating Scale [DRS]) and noncognitive functional assessments (Clinical Dementia Rating scale, sum of boxes [CDR-SOB] and motor impairment by Unified Parkinson Disease Rating Scale part III [UPDRS-III]). Neuropsychological evaluations included the Auditory Verbal Learning Test (AVLT) for memory, the Boston Naming Test (BNT) for object naming, the Trail Making Test, part A (TMT-A) for divided attention, and the Rey Complex Figure (RCF) test for visual-perceptual processing.

The study was approved by the Mayo Clinic institutional review board, and informed consent on participation was obtained from every participant or an appropriate surrogate. The study followed Strengthening the Reporting of Observational Studies in Epidemiology (STROBE) reporting guideline.

### Imaging Study

Baseline and follow-up PiB PET imaging was performed on PET-computed tomography systems operating in a 3-dimensional mode (GE Medical Systems). Scans consisted of four 5-minute dynamic frames acquired from 40 to 60 minutes after injection of PiB; detailed descriptions have been published elsewhere.^15,20^ For anatomic segmentation and labeling of PiB PET images, 3-dimensional, high-resolution, magnetization-prepared rapid gradient echo T1-weighted magnetic resonance imaging (MRI) scans, performed during the same visit cycle as the PiB PET, were acquired with a 3-T MRI scanner with 1 mm^3 ^resolution (GE Medical Systems).^21^ Baseline and follow-up MRI images were automatically segmented and bias corrected using unified segmentation^22^ in statistical parametric mapping 12. We rigidly aligned PET images to MRI images, using statistical parametric mapping 12 (baseline-to-baseline and follow-up–to–follow-up), and MRI segmentations were used to perform 2-class partial volume correction.^23^ For consistency, we also performed analyses with no partial volume correction of PiB SUVR. Regions were automatically located using advanced normalization tools^24^ with the Mayo Clinic Adult Lifespan Template.^25,26^ For each PiB image, PiB uptake was calculated as the SUVR in a standard composite region consisting of voxels in the parietal, posterior cingulate, precuneus, prefrontal, orbitofrontal, temporal, and anterior cingulate cortices.^15^ To maximize the reliability and plausibility of measurements, we used 2 reference regions: 1 for baseline PiB SUVR and 1 for longitudinal change in PiB SUVR. For the baseline PiB SUVR measurement, we used a standard cerebellar crus reference region.^27^ To measure the change in PiB SUVR, we used a composite reference region of eroded supratentorial white matter, whole cerebellum, and pons; this technique was developed by our group, has been extensively tested and compared with multiple alternative approaches, and has been shown to improve reliability and plausibility for serial measurements compared with cross-sectional approaches.^20^

### Statistical Analysis

Demographic, clinical, and cognitive characteristics of participants with probable DLB and CU participants at baseline were summarized using means with SDs or proportions. A log transformation or a square root transformation was performed to normalize the distribution of baseline PiB SUVR, MMSE score, and CDR-SOB score. Continuous variables were compared between probable DLB and CU groups using analysis of variance with a random block design with an added predictor to account for matching. The change in PiB SUVR for probable DLB and CU groups was constructed from partial volume-corrected serial PiB SUVR. Changes in PiB SUVR and in clinical and cognitive measures were annualized. We chose generalized additive models (GAMs) with 95% CIs to model the change in PiB SUVR as a function of baseline PiB SUVR. We used 4-*df *penalized splines in GAMs as our primary analysis to estimate the shapes of change in PiB SUVR vs baseline PiB SUVR for probable DLB and CU groups separately. Subsequently, we tested for a type of interaction between group (probable DLB or CU) and change in PiB SUVR by fitting fixed 4-*df* regression splines (to control the smooths and produce nested models) within each group and then by fitting a 4-*df* regression spline without differentiating the groups. We used an approximate F test from the analysis of deviance table comparing the models to test the interaction. We used GAMs to estimate the cumulative PiB SUVR as a function of time in years in the probable DLB and CU groups; GAMs accounted for matching between the groups. We used linear regression models to determine the association of baseline PiB SUVR and rate of change in PiB SUVR with rate of change in measures of clinical and cognitive decline. We reported results of models without adjustment for any covariates. We investigated regression models, adjusting for combinations of age, sex, education, and *APOE* e4 carrier status but found that no covariates were statistically significant nor did inclusion of the covariates produce qualitatively different results for PiB SUVR or change in PiB SUVR. Finally, in the probable DLB group, we estimated sample size for a hypothetical anti–β-amyloid clinical trial in patients with probable DLB. Mixed-effect models and the jackknife-based resampling method were used to estimate the sample sizes expressed as mean values with asymptotic confidence intervals. Change in PiB SUVR, CDR-SOB score, DRS score, and MMSE score were used for these calculations, assuming 1-sided tests, 80% power, α = 0.05, and readings at 12, 18, and 24 months of follow-up. Analyses were performed using SAS statistical software version 9.4 (SAS Institute) and R statistical software version 3.1.1 (R Foundation for Statistical Computing) with *P* < .05 considered statistically significant. All tests were 2-tailed, except for tests for sample size estimates, which were were 1-tailed.

## Results

### Baseline Cohort Characteristics

Baseline characteristics of participants in the probable DLB and CU groups, matched on age, sex, and *APOE *e4 status, are listed in Table 1. In total, 175 participants were evaluated. Of these, 35 (20.0%) had probable DLB, with mean (SD) age of 69.6 (7.3) years; 16 (45.7%) were *APOE *e4 carriers; and 31 (88.6%) were men. A total of 140 CU participants (80.0%) were matched on age (mean [SD] age 69.7 [7.2] years), *APOE *e4 status (64 [45.7%] carriers), and sex (124 [88.6%] men) to patients with probable DLB. Dementia severity of participants with probable DLB was mild based on MMSE, DRS, and CDR-SOB scores. Mean (SD) baseline PiB SUVR, reported with partial volume correction, was higher among participants with probable DLB than among CU participants (1.58 [0.41] vs 1.36 [0.22]; *P* < .001; range, 1.17-2.57 vs 1.11-2.36). We obtained similar results on baseline PiB SUVR and findings in this study when we analyzed PiB SUVR data with no partial volume correction (mean [SD] baseline PiB SUVR 1.44 [0.36] vs 1.26 [0.20]; *P* < .001; range, 1.05-2.23 vs 1.01-2.21). The interval between baseline and follow-up visit was shorter among the probable DLB group than the CU group because of recruitment from 2 sources; therefore, change in PiB SUVR and changes in clinical and cognitive measures were annualized. Compared with patients with probable DLB who did not carry *APOE *e4, *APOE *e4 carriers had higher mean (SD) baseline PiB SUVR (1.40 [0.27] vs 1.79 [0.46]; *P* = .005) and lower mean (SD) UPDRS-III motor score (11.1 [5.5] vs 6.5 [5.7]; *P* = .02). In clinical and cognitive measures and frequencies of probable DLB, *APOE *e4 carriers vs noncarriers did not differ (ie, all *P* > .05). We did not examine differences in the change in PiB SUVR between participants with probable DLB who were *APOE *e4 carriers vs noncarriers because of relatively small subgroups.

**Table 1.** Participants’ Baseline Characteristics

Characteristic	Mean (SD)		*P* Value^a^
	CU Participants (n = 140)	Patients With Probable DLB (n = 35)	
Men, No. (%)	124 (88.6)	31 (88.6)	>.99
Age, y	69.7 (7.2)	69.6 (7.3)	.68
*APOE* e4 carrier, No. (%)	64 (45.7)	16 (45.7)	>.99
Education, y	15.3 (2.4)	15.7 (2.9)	.44
Interscan interval, y	2.4 (1.0)	1.2 (0.4)	<.001
PiB SUVR			
Baseline, mean (SD) [range]	1.36 (0.22) [1.11-2.36]	1.58 (0.41) [1.17-2.57]	<.001^b^
Slope, baseline to follow-up	0.016 (0.024)	0.020 (0.037)	.45
CDR-SOB score^c^	0.0 (0.2)	3.4 (1.8)	<.001^b^
MMSE score^c^	28.5 (1.1)	24.3 (4.7)	<.001^b^
UPDRS-III motor score^d^	0.4 (1.2)	9.1 (6.0)	<.001
AVLT, delayed recall score^e^	8.2 (2.9)	3.2 (3.4)	<.001
TMT-A score^f^	33.6 (9.0)	69.0 (38.4)	<.001
BNT score^g^	NA	25.3 (4.7)	NA
RCF copy, total score^g^	NA	17.9 (10.5)	NA
DRS score^h^	NA	128.6 (8.9)	NA
Visual hallucination, No. (%)^i^	NA	17 (50.0)	NA
Fluctuations, No. (%)^i^	NA	22 (64.7)	NA
Parkinsonism, No. (%)^i^	NA	29 (85.3)	NA
RBD, No. (%)^i^^,^^j^	NA	33 (97.1)	NA
Cognitive impairment, y^i^	NA	5.58 (3.32)	NA

Abbreviations: *APOE*, apolipoprotein; AVLT, Auditory Verbal Learning Test; BNT, Boston Naming Test; CDR-SOB, Clinical Dementia Rating Scale, sum of boxes; CU, cognitively unimpaired; DLB, dementia with Lewy bodies; DRS, Dementia Rating Scale; MMSE, Mini-Mental State Examination; PiB SUVR, carbon-11 labeled Pittsburgh compound B, standardized uptake value ratio; RCF, Rey Complex Figure; RBD, REM Sleep Behavior Disorder; TMT-A, Trail Making Test, part A; UPDRS-III, Unified Parkinson Disease Rating Scale, part III.

^a^ *P* values for differences between groups came from an analysis of variance using a random block design with an added predictor for the matching ID.

^b^ Either a log transformation or square root transformation was performed to normalize the distribution.

^c^ Data missing for 1 CU participant.

^d^ Data missing for 1 CU participant and 1 participant with probable DLB.

^e^ Data missing for 1 CU participant and 6 participants with probable DLB.

^f^ Data missing for 1 CU participant and 2 participants with probable DLB.

^g^ Data missing for 5 participants with probable DLB.

^h^ Data missing for 6 participants with probable DLB.

^i^ Data missing for 1 participant with probable DLB.

^j^ A total of 25 of 34 patients (73.5%) with probable DLB had probable RBD confirmed by polysomnography; 8 (23.5%) had possible RBD confirmed by Mayo Clinic Sleep Questionnaire^18^; and 1 (2.9%) did not have RBD.

### Trajectories of Change in PiB SUVR

**Figure 1.**
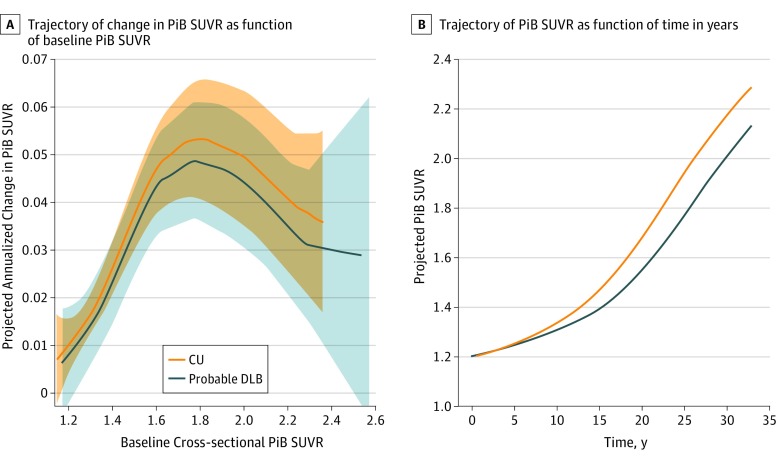
Trajectories of Change in Carbon-11 Labeled Pittsburgh Compound B Standardized Uptake Value Ratio (PiB SUVR) and Baseline PiB SUVR A, Regardless of clinical group, change in PiB SUVR increases, peaks at a baseline PiB SUVR of approximately 1.8, and then decreases, forming an inverted U–shaped curve. Change in PiB SUVR did not differ between the probable dementia with Lewy bodies (DLB) and cognitively unimpaired (CU) groups in the shape or vertical shift between the trajectories; confidence bands, indicated by shaded areas, largely overlap. The widening of the confidence bands on the right side of the panel reflects the lower number of participants (n = 11) with higher baseline PiB SUVR values (ie, >1.7). B, The inverted U–shaped curves were integrated into the sigmoid-shaped trajectory of cumulative PiB SUVR as a function of time in years.

Change in PiB SUVR by baseline PiB SUVR did not differ between the probable DLB and CU groups (Figure 1A); the regression-based smooth curves for rate of change in PiB SUVR did not differ between DLB and CU (*P* = .59). Moreover, we observed no difference in the shape (vertical shift) of trajectories between the probable DLB and CU groups (regression spline model, approximate *P* = .07; penalized spline model, coefficient [SE] 0.007 [0.006]; *P* = .22) (Figure 1A). The association between change in PiB SUVR and baseline PiB SUVR was nonlinear (test of linearity, *P* < .001) in both PDLB and CU groups. In both probable DLB and CU groups, change in PiB SUVR accelerated at lower baseline PiB SUVR, peaked at a PiB SUVR of approximately 1.8, and then decelerated at higher baseline PiB SUVR, forming an inverted U–shaped curve as a function of baseline PiB SUVR. Subsequently, the associations of change in PiB SUVR as a function of baseline PiB SUVR were integrated into PiB SUVR as a function of time associations in probable DLB and CU groups (ie, the cumulative density function) (Figure 1B). The integral association of PiB SUVR by time rendered sigmoid-shaped trajectories for both probable DLB and CU groups (Figure 1B).

### Association of Baseline and Change in PiB SUVR With Clinical and Cognitive Decline in Patients With Probable DLB

**Figure 2.**
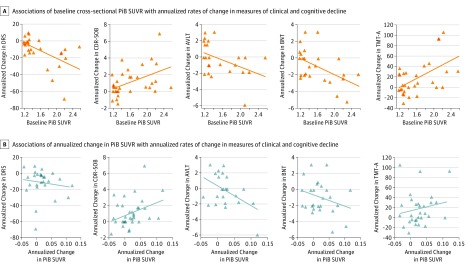
Rate of Change in Clinical and Cognitive Measures by Baseline Carbon-11 Labeled Pittsburgh Compound B Standardized Uptake Value Ratios (PiB SUVR) and Change in PiB SUVR Among Patients with Probable Dementia with Lewy Bodies A, Scatterplots show significant associations of the baseline cross-sectional PiB SUVR with the annualized rates of change in measures of clinical and cognitive decline in patients with probable DLB. B, Scatterplots show associations of change in PiB SUVR with changes in measures of clinical and cognitive decline; associations with change in Clinical Dementia Rating, sum of boxes (CDR-SOB) score and Auditory Verbal Learning Test (AVLT), delayed recall are significant. The estimates for these associations are from simple linear regression models (Table 2). BNT indicates Boston Naming Test; DRS, Dementia Rating Scale; and TMT-A, Trail Making Test, part A.

In patients with probable DLB, the associations of baseline PiB SUVR and change in PiB SUVR with measures of clinical progression are summarized in Table 2 and Figure 2. Higher baseline PiB SUVR was associated with a greater longitudinal decline, as measured by the DRS (regression coefficient [SE], −22.40 [6.53]; *P* = .002; *R*^2^ = 0.312), the CDR-SOB (regression coefficient [SE], 1.90 [0.63]; *P* = .005; *R*^2^ = 0.215), the AVLT, delayed recall (regression coefficient [SE], −2.09 [0.95]; *P* = .04; *R*^2^ = 0.182), the BNT (regression coefficient [SE], −2.39 [0.84]; *P* = .009; *R*^2^ = 0.245), and the TMT-A (regression coefficient [SE], 43.43 [12.96]; *P* = .002; *R*^2^ = 0.286). Similarly, greater change in PiB SUVR was associated with greater decline as measured by the CDR-SOB (regression coefficient [SE], 16.17 [7.47]; *P* = .04; *R*^2^ = 0.124) and the AVLT, delayed recall (regression coefficient [SE], −25.05 [10.04]; *P* = .02; *R*^2^ = 0.221). Baseline PiB SUVR and change in PiB SUVR were not associated with changes in MMSE score, UPDRS-III score, or visual-perceptual processing (Table 2).

**Table 2.** Associations of Baseline PiB SUVR and Change in PiB SUVR With Clinical and Cognitive Decline in Probable Dementia with Lewy Bodies

Change in Measure	Regression Coefficient (SE)^a^	*P* Value	*R*^2^
**Baseline PiB SUVR**			
DRS	–22.40 (6.53)	.002	0.312
CDR-SOB	1.90 (0.63)	.005	0.215
MMSE	–2.25 (1.78)	.22	0.046
UPDRS-III	–0.93 (1.74)	.60	0.009
AVLT	–2.09 (0.95)	.04	0.182
BNT	–2.39 (0.84)	.009	0.245
TMT-A	43.43 (12.96)	.002	0.286
ROCFT	–4.26 (3.76)	.27	0.047
**Change in PiB SUVR**			
DRS	–62.09 (86.67)	.48	0.019
CDR-SOB	16.17 (7.47)	.04	0.124
MMSE	–28.40 (19.78)	.16	0.059
UPDRS-III	6.66 (19.39)	.73	0.004
AVLT	–25.05 (10.04)	.02	0.221
BNT	–13.81 (9.80)	.17	0.074
TMT-A	153.42 (167.73)	.37	0.029
ROCFT	30.08 (39.88)	.46	0.021

Abbreviations: AVLT, Auditory Verbal Learning Test, delayed recall; BNT, Boston Naming Test; CDR-SOB, Clinical Dementia Rating, sum of boxes; DRS, Dementia Rating Scale; MMSE, Mini-Mental State Examination; PiB SUVR, carbon-11 labeled Pittsburgh compound B, standardized uptake value ratio; TMT-A, Trail Making Test, part A; UPDRS-III, Unified Parkinson Disease Rating Scale, part III, motor score.

^a^ Regression coefficients for these associations are from simple linear regression models.

The nature of the selection of the CU participants resulted in a restricted range of change in cognition and clinical scales. For example, only 8 CU participants (5.7%) had nonzero values for change in CDR-SOB score. Thus, the findings from only 8 influential participants would have to be interpreted with extreme caution. In addition, since we selected CU participants to match patients with probable DLB on age, sex, and *APOE* e4 status, we could only make inferences about this CU sample, which does not fully represent the CU population.

### Sample Size Estimates for Hypothetical Clinical Trial in DLB

The sample size estimates for a hypothetical clinical trial in patients with DLB showed that using the change in PiB SUVR to measure therapeutic effect would require the smallest sample size. Change in PiB SUVR was followed by change in CDR-SOB score, whereas using the measurements of changes in DRS and MMSE scores would require larger samples (Table 3).

**Table 3.** Sample Size Estimates for Hypothetical Clinical Trial in Dementia with Lewy Bodies^a^

Measure	Participants, No. (95% CI)							
Follow-up, mo	12	12	18	18	24	24	36	
Reduction in slope, %	25	50	25	50	25	50	25	50
PiB SUVR	602 (521-682)	151 (131-170)	258 (224-292)	65 (57-73)	151 (131-171)	38 (33-43)	61 (53-69)	16 (14-17)
CDR-SOB	768 (655-882)	193 (164-221)	328 (280-377)	83 (71-95)	193 (164-222)	49 (42-56)	77 (66-89)	20 (17-22)
DRS	867 (735-1000)	215 (181-251)	370 (309-431)	94 (79-108)	218 (185-250)	55 (46-63)	87 (75-100)	22 (19-26)
MMSE	1583 (1262-1904)	397 (321-472)	681 (543-820)	170 (138-203)	395 (313-477)	99 (79-118)	159 (127-190)	40 (32-48)

Abbreviations: CDR-SOB, Clinical Dementia Rating, sum of boxes; DRS, Dementia Rating Scale; MMSE, Mini-Mental State Examination; PiB SUVR, carbon-11 labeled Pittsburgh compound B, standardized uptake value ratio.

^a^ Slope estimates and variances are from mixed models. Sample sizes are estimated using jackknife resampling as mean values along with asymptotic confidence intervals.

## Discussion

In this longitudinal cohort PiB PET study, we determined the trajectories of change in PiB SUVR in patients with mild probable DLB compared with CU participants, matched on demographic variables and *APOE *e4 status. The trajectories of change in PiB SUVR did not differ between probable DLB and CU groups. In both groups, the trajectories were nonlinear, with an initial acceleration at lower baseline PiB SUVR followed by a deceleration at higher baseline PiB SUVR. The integral association between cumulative PiB SUVR and time showed a sigmoid-shaped functional form in both probable DLB and CU groups, very similar to the trajectories reported in AD continuum cohorts, which included CU participants with a range of baseline PiB SUVRs.^13,14,15^ Furthermore, the rate of clinical progression in probable DLB was associated with both baseline PiB SUVR and change in PiB SUVR. We showed that measuring change in PiB SUVR and change in CDR-SOB score would require a smaller sample size in a hypothetical clinical trial among patients with probable DLB. Altogether, our findings suggest that measuring change in PiB SUVR is a valid biomarker of longitudinal β-amyloid accumulation in individuals with probable DLB and that progression of β-amyloid pathology in probable DLB is associated with functional and cognitive decline.

We compared change in PiB SUVR between participants with probable DLB and CU participants who were matched by age, sex, and *APOE *e4 status. We hypothesized that such matching could allow for an indirect evaluation of the effect of α-synuclein on the change in PiB SUVR in participants with probable DLB. Interestingly, we found that change in PiB SUVR in the probable DLB group did not diverge from the CU group. However, the trajectories of change in PiB SUVR seen in our study closely resembled the trajectories of change in PiB SUVR in previous longitudinal studies on change in PiB SUVR among CU patients, patients with MCI, and patients with AD.^13,14,15^ These similarities across large cohorts and studies would suggest a relatively uniform progression of β-amyloid pathology with respect to baseline β-amyloid load in various neurodegenerative syndromes (ie, AD and DLB) and individuals with no cognitive impairment.

We note that the primary underlying pathology contributing to cognitive impairment in probable DLB patients is α-synuclein, with additional β-amyloid, NFT-tau, and possibly other pathologies, such as vascular disease or TAR DNA-binding protein-43. There is growing evidence of complex interactions between α-synuclein, β-amyloid, and NFT-tau,^4,28,29^ such that individuals with higher α-synuclein levels also tend to have higher β-amyloid and NFT-tau burdens. However, our findings suggest that the likely presence of α-synuclein in patients with mild probable DLB does not significantly alter the trajectory of β-amyloid accumulation as measured by PET.

The associations of baseline PiB SUVR with clinical and cognitive impairment have been ambiguous in probable DLB,^12^ which may be because of discrepancies in study design, small sample sizes of generally cross-sectional cohorts, and discrepancies in the interpretation of findings because observing an association is not equal to finding a causal association. Many studies combined patients with probable DLB, Parkinson disease dementia, or even MCI with Parkinson disease in 1 group. Some reported an association of higher PiB SUVR with lower MMSE scores,^30^ worse semantic memory,^30,31^ or lower CDR scores,^32^ whereas others did not find an association with MMSE^33^ or CDR scores.^21^ A study performed by our group^34^ observed an association of higher baseline PiB SUVR with worsening in CDR-SOB score over time. In the current study, we showed associations of baseline PiB SUVR with measures of longitudinal clinical and cognitive decline in patients with DLB. We found that a higher baseline PiB SUVR was associated with a more rapid decline as measured by DRS, CDR-SOB, AVLT, BNT, and TMT-A. Moreover, longitudinally, a greater change in PiB SUVR was associated with greater changes in CDR-SOB and AVLT scores. Thus, these 2 measures may be more sensitive and optimal for monitoring the cooccurrence of β-amyloid progression and clinical progression in probable DLB. The association of memory decline with PiB SUVR in probable DLB is interesting because, early in the AD continuum, many studies did not confirm associations of baseline PiB SUVR or change in PiB SUVR with memory decline.^35,36^ This could be owing to floor effect in AD and MCI studies, in which baseline memory performance is already moderately to severely impaired, but in DLB, baseline memory scores are less impaired. Aside from methodological issues, a potential biological explanation has been that β-amyloid alone is insufficient to influence cognitive impairment directly and rather constitutes an early event causing a chain of downstream pathologic changes leading to cognitive decline.^35,37,38^ We have shown that a higher PiB SUVR in patients with probable DLB was associated with higher fluoride-18 flortaucipir (AV-1451) uptake.^39^ It remains to be seen whether the associations of baseline PiB SUVR and change in PiB SUVR with clinical and cognitive decline in probable DLB are direct effects of the progression of β-amyloid accumulation or whether it is the progression of α-synuclein or NFT-tau that influences cognitive decline, thus making the association of β-amyloid progression with clinical and cognitive decline indirect.

There was no association of PiB SUVR or change in PiB SUVR with changes in MMSE score, UPDRS-III score, or RCF-measured visual-perceptual performance. A potential explanation is lower statistical power or relatively narrow range of values in a probable DLB group of this size. Additionally, cognitive fluctuations may contribute to both short-term and long-term variability in clinical and cognitive evaluations. Moreover, the MMSE might not be an optimal measure of global cognitive decline in probable DLB,^40^ although some studies have suggested otherwise.^41^ Most importantly, these clinical and cognitive measures may be influenced by other pathologies, such as NFT-tau or α-synuclein, or by other neurologic and functional factors, such as mood or daytime sleepiness.

Sample size calculations for a hypothetical clinical trial in patients with probable DLB showed that measuring change in PiB SUVR followed by change in CDR-SOB score required the smallest sample size compared with the most often–used global cognitive and functional measures. Favorable sample size estimates using change in CDR-SOB score may again suggest that global functional measures may be more optimal for tracking overall impairment in probable DLB and may track better with complex symptoms, such as cognitive, motor, sleep-related, affective, and psychiatric symptoms. Conversely, a large sample size by change in MMSE score indicated that the MMSE may not be an optimal measure for global cognitive decline in probable DLB in a clinical trial setting.

### Limitations

Our study has some limitations. Although this longitudinal study sample was larger than most cross-sectional β-amyloid PET studies among individuals with probable DLB, it may still not have the sufficient power to detect subtle associations or conduct subgroup analyses, such as change in PiB SURV by *APOE *e4 status or by sex. The differences in change in PiB SUVR between CU *APOE *e4 carriers vs noncarriers were previously investigated^42^ but need to be investigated among individuals with probable DLB. A recent meta-analysis did not show greater prevalence of β-amyloid pathology by PET in women vs men within the AD continuum,^43^ but the sex effects need to be investigated further in probable DLB. Furthermore, CU participants may have various subthreshold pathologies owing to their population-based origin.^44^ Approximately 30% of CU participants have elevated baseline PiB SUVR.^43^ Some of them develop cognitive impairment and dementia,^35^ whereas others remain without cognitive impairment. It is likely that some of the CU participants in this study will later develop cognitive impairment. To mitigate this, CU participants had to remain clinically unimpaired during the follow-up period.

## Conclusions

In this cohort study, the sigmoid trajectory of cumulative PiB SUVR by time observed in patients with probable DLB was consistent with the trajectories in the AD continuum, including the CU participants with lower baseline PiB SUVR. This finding suggests that, at sufficiently high baseline PiB SUVR, PiB uptake would reach equilibrium. This has potential implications for the timing of potential anti–β-amyloid strategies in probable DLB. Whereas the consequences of anti–β-amyloid approaches among patients with probable DLB are unknown at this time, associations of PiB SUVR and change in PiB SUVR with clinical and cognitive decline suggest that anti–β-amyloid strategies may have a place in clinical trials involving patients with probable DLB. However, how an anti–β-amyloid treatment would affect the progression of α-synuclein and NFT-tau in probable DLB remains to be seen. Because of the interactions among β-amyloid, α-synuclein, and NFT-tau,^3,4^ it is possible that targeting β-amyloid alone might contribute to overall pathologic progression and functional improvement in probable DLB patients. However, owing to the heterogeneity and complexity of underlying proteinopathies and clinical symptoms in probable DLB, individualized combination therapies with acetyl-cholinesterase inhibitors,^45^ lifestyle interventions, treatment of age-related comorbidities, and anti-tau treatments will need to be considered.
